# *PAX5* Alterations in a Consecutive Childhood B-Cell Acute Lymphoblastic Leukemia Cohort Treated Using the ALL IC-BFM 2009 Protocol

**DOI:** 10.3390/cancers16061164

**Published:** 2024-03-15

**Authors:** Klementina Črepinšek, Nika Klobučar, Tine Tesovnik, Robert Šket, Barbara Jenko Bizjan, Jernej Kovač, Marko Kavčič, Tomaž Prelog, Lidija Kitanovski, Janez Jazbec, Maruša Debeljak

**Affiliations:** 1 Clinical Institute for Special Laboratory Diagnostics, University Children’s Hospital, University Medical Centre Ljubljana, Vrazov trg 1, 1000 Ljubljana, Slovenia; klementina.crepinsek@kclj.si (K.Č.);; 2 Department of Oncology and Haematology, University Children’s Hospital, University Medical Centre Ljubljana, Bohoričeva ulica 20, 1000 Ljubljana, Slovenia; 3 Faculty of Medicine, University of Ljubljana, Vrazov trg 2, 1000 Ljubljana, Slovenia

**Keywords:** childhood B-cell acute lymphoblastic leukemia, *PAX5* genetic alterations, prognostic significance

## Abstract

**Simple Summary:**

B-cell acute lymphoblastic leukemia is genetically diverse, with one of the most commonly altered genes being *PAX5*. Recently, alterations in *PAX5* have been identified not merely as secondary events, but also as the oncogenic drivers, with newly defined genetic subtypes like PAX5alt and PAX5 P80R. Studying a consecutive cohort of 99 B-ALL patients, we found *PAX5* alterations in over a third of patients, mostly involving copy number variations. Seven of our patients exhibited the PAX5alt genetic profile and one carried the P80R variant, and these patients showed intermediate outcomes. We also discovered that patients within the hyperdiploid group that carried extra copies of *PAX5* did not respond as well to the treatment. Overall, our study highlights widespread *PAX5* alterations affecting treatment response, particularly in specific genetic subtypes, underlining the need to identify these alterations for improved treatment strategies.

**Abstract:**

In this study, we aimed to identify patients within our B-ALL cohort with altered *PAX5*. Our objective was to use a comprehensive analysis approach to characterize the types of genetic changes, determine their origin (somatic/germline), and analyze the clinical outcomes associated with them. A consecutive cohort of 99 patients with B-ALL treated at the Children’s Hospital of the UMC Ljubljana according to the ALL IC-BFM 2009 protocol was included in our study. We used RNA sequencing data for gene expression analysis, fusion gene detection and single nucleotide variant identification, multiplex-ligation dependent probe amplification for copy number variation assessment, and Sanger sequencing for germline variant detection. *PAX5* was impacted in 33.3% of our patients, with the genetic alterations ranging from CNVs and rearrangements to SNVs. The most common were CNVs, which were found in more than a third of patients, followed by point mutations in 5.2%, and gene rearrangements in 4.1%. We identified eight patients with a *PAX5*-associated genetic subtype that were previously classified as “B-other”, and they showed intermediate outcomes. We showed higher minimal residual disease values at the end of induction and poorer event-free survival in hyperdiploid cases carrying duplications in *PAX5* compared to other hyperdiploid cases. We also report an interesting case of a patient with *PAX5::FKBP15* and a pathogenic variant in *PTPN11* who underwent an early relapse with a monocytic switch. In conclusion, this study provides valuable insights into the presence, frequency, and prognostic significance of diverse *PAX5* alterations in B-ALL patients, highlighting the complexity of genetic factors and their impact on patient outcomes.

## 1. Introduction

Childhood B-cell acute lymphoblastic leukemia (B-ALL) remains a challenging hematological malignancy, necessitating ongoing research to identify novel markers for improved treatment outcomes. Among the genes garnering significant attention in recent years, *PAX5* stands out as a critical regulator of B-cell development. Understanding its genetic background and underlying mechanisms has become paramount to unravelling the pathogenesis of B-ALL and exploring its clinical implications.

The *PAX5* gene belongs to the PAX gene family, encompassing transcription factors that control several developmental processes. *PAX5* exhibits a predominant expression in B-cell precursors and plays a central role in their commitment, differentiation, and maintenance [1]. The protein PAX5 contains 391 amino acids and is divided into the N-terminus domain, responsible for binding to specific DNA sequences, the octapeptide and partial homeodomain in the center, involved in protein–protein interactions, and the transactivation and inhibitory domains at the C-terminus, which regulate gene expression. The functional integrity of the C-terminal domain stands out as a pivotal factor in B-cell development. The disruption of its normal function has been found to significantly promote the pathogenesis of leukemias, particularly B-cell ALL [2,3].

Numerous abnormalities associated with *PAX5* have been identified, including gene rearrangements, sequence variants, focal intragenic amplifications, and deletions [4,5]. Comprehensive genomic studies have highlighted *PAX5* as the most commonly altered gene in B-ALL, with the alterations being present in approximately one-third of pediatric and adult cases [5,6,7]. Recent evidence suggests that alterations in *PAX5* are not merely secondary events in B-ALL, but often serve as the primary drivers of leukemogenesis [7]. Moreover, *PAX5* variants are also sometimes present in the germline, contributing to B-ALL susceptibility [8,9]. This highlights the significance of examining both somatic and germline alterations in *PAX5*, expanding our understanding of disease predisposition and the interplay between genetic and inherited factors.

In light of the diverse range of genetic alterations involving *PAX5*, two distinct B-ALL genetic subtypes have been defined: PAX5 P80R and PAX5alt. The PAX5 P80R subtype has demonstrated variable prognostic effects across different cohort studies, indicating the need for further investigation to accurately elucidate its clinical implications. Conversely, patients with the PAX5alt subtype exhibit consistently poorer outcomes, especially in the presence of co-occurring *IKZF1* deletions [7,10]. 

With the identification and definition of the two novel genetic subtypes involving *PAX5* and identifying *PAX5* as one of the key players also in Ph-like ALL [7,11,12], there is a growing interest in investigating the various changes and underlying mechanisms, as well as their implications for patient prognoses. Therefore, in this study, we aimed to identify patients within our B-ALL cohort with altered *PAX5* using a comprehensive analysis approach, characterize the types of genetic changes, determine their somatic or germline origin, and analyze the clinical outcomes associated with these alterations, ultimately aiming to improve patient outcomes and provide a better understanding of the role of *PAX5* in leukemogenesis.

## 2. Materials and Methods

### 2.1. Patients and Materials

A consecutive cohort of 99 patients with B-ALL that were diagnosed between January 2012 and December 2020 and treated at the Children’s Hospital of the University Medical Centre Ljubljana according to the ALL IC-BFM 2009 protocol was included in our study. Diagnoses were established following standard clinical, cytomorphological, cytogenetic, molecular genetic, and immunological criteria. Minimal residual disease (MRD) was measured by flow cytometry according to the established laboratory protocol. 

We collected bone marrow for 92 patients and peripheral blood samples for 7 patients as part of the diagnostic procedure before starting treatment. For 7 of these 99 patients, we also collected samples to test for the presence of germline variants, buccal swab samples from 5 patients, and peripheral blood samples during remission for 2 patients.

For 4 patients, there was not sufficient material available to perform the multiplex ligation-dependent probe amplification (MLPA) assay, and for an additional 3, the assay failed due to poor sample quality. Therefore, MLPA data analysis included 92 patients from the cohort. For RNA sequencing, there was not sufficient material for two patients, therefore the analysis was carried out for 97 samples.

The study was conducted according to the guidelines of the Declaration of Helsinki and approved by the Republic of Slovenia National Medical Ethics Committee on 10 March 2011 and 15 March 2022 (reference number KME 51/03/11 and KME 0120-17/2022/6). Informed consent was obtained from all subjects involved in the study. Written informed consent was obtained from the patients or parents to publish this paper or any other anonymous data and results.

### 2.2. DNA and RNA Isolation

The DNA from bone marrow and peripheral blood samples was isolated using the FlexGene DNA kit according to the manufacturer’s instructions (Qiagen, Hilden, Germany). The DNA from buccal swab samples was isolated using the QIAamp DNA Mini Kit according to the manufacturer instructions (Qiagen, Hilden, Germany). The extracted DNA was stored at 4 °C.

The bone marrow and peripheral blood samples for RNA isolation were stored in the TRIzol^®^ reagent (Thermno Fisher Scientific, Waltham, MA, USA). After the addition of chloroform and phase separation, the Direct-zol™ RNA MiniPrep Kit was used for RNA extraction (Zymo, Irvine, CA, USA). The extracted RNA was stored at –70 °C. 

### 2.3. RNA Sequencing and Data Analysis

Bone marrow and peripheral blood samples at the time of diagnosis for transcriptome analysis were prepared using the NEBNext Globin & rRNA Depletion Kit Human/Mouse/Rat (New England Biolabs, Ipswich, MA, USA) and NEBNext Ultra II Directional RNA Library Prep Kit for Illumina (New England Biolabs, Ipswich, MA, USA). We sequenced the samples with 100 million reads per sample using a NovaSeq 6000 system (Illumina, San Diego, CA, USA).

The RNA expression was determined by bioinformatic analysis of the data based on the SnakePipes tool [13]. Shortly, the Cutadapt tool [14] was used to remove adapter sequences and low-quality sequences. We used the STAR tool [15] to align the reads to the human reference genome (GRCh38) and featureCounts [16] to count gene expression levels. 

To determine the B-ALL genetic subtype for each patient, we used two R-based tools, the gene expression classifier tool ALLCatchR [17] and MD-ALL [18]. ALLCatchR uses only a gene expression read count as the input and distinguishes between 21 BCP-ALL molecular subtypes (BCL2/MYC, CDX2/UBTF, CEBP, DUX4, ETV6::RUNX1, ETV6::RUNX1-like, HLF, hyperdiploid, iAMP21, IKZF1 N159Y, KMT2A, low hypodiploid, MEF2D, near haploid, NUTM1, PAX5 P80R, PAX5alt, Ph-like, Ph-pos, TCF3::PBX1, ZNF384). MD-ALL takes the read counts, VCF files, and raw outputs of fusion callers (FusionCatcher and/or Cicero) as input files and classifies B-ALL into 26 subtypes (BCL2/MYC, CDX2/UBTF, CRLF2 (non-Ph-like), DUX4, ETV6::RUNX1, ETV6::RUNX1-like, HLF, hyperdiploid, iAMP21, IKZF1 N159Y, KMT2A, KMT2A-like, low hypodiploid, low hyperdiploid, MEF2D, near haploid, NUTM1, PAX5 P80R, PAX5::ETV6, PAX5alt, Ph, Ph-like, TCF3::PBX1, ZEB2/CEBP, ZNF384, ZNF384-like).

Gene fusions were detected from RNA sequencing data using nf-core’s pipeline rnafusion on default settings (version 2.3.4) [19]. This pipeline uses several tools for fusion detection (Arriba [20], Pizzly [21], STAR-fusion [22], Squid [23], and FusionCatcher [24]) and combines the results from all tools in a joint report for each sample. Additionally, we used CICERO [25] for fusion detection, as the use of this tool is needed for a complete subtype analysis using the MD-ALL tool. When needed, the data were manually inspected to confirm the presence of the fusion transcripts.

All sequences were aligned to the human reference genome (GRCh38) and variants were detected using the nf-core rnavar pipeline (version 1.0.0) [26] for RNA sequencing data. This pipeline aligns reads to the reference genome with STAR. Variant calling was carried out according to the Genome Analysis Toolkit (GATK) best practices for variant calling [27]. Annotation was carried out with Annovar [28]. We analyzed 549 cancer-related genes from the UCSF500 Cancer gene panel (accessed on 31 August 2023 on https://genomics.ucsf.edu/UCSF500) (see Supplementary Table S1). Mutations were required to have a read depth of at least 30 and a VAF > 10%. Population variants were excluded based on gnomAD and 1000G annotations (variants with allele frequency ≤ 0.01 were kept for analysis). We carried out a further selection of the variants with the prediction tools UMD Predictor (probable pathogenic and pathogenic), PolyPhen 2 HumDiv (possibly damaging and probably damaging), Provean (damaging), and CADD score (>15).

### 2.4. Multiplex Ligation-Dependent Probe Amplification for CNV Detection

DNA was analyzed for copy number alterations using the SALSA MLPA P335 ALL-IKZF1 probemix, according to the manufacturer’s instructions (MRC Holland, Amsterdam, The Netherlands). The P335 probemix allows for the detection of deletions and duplications in B-cell differentiation and cell cycle control genes (*IKZF1*, *CDKN2A/B*, *PAX5*, *EBF1*, *ETV6*, *BTG1*, and *RB1*), as well as in genes from the X/Y PAR1 region (*CRLF2*, *CSF2RA*, *SHOX*, *IL3RA*, and *P2RY8*). It also contains 13 reference probes that function as internal controls. MLPA reactions were carried out on a 96-well PCR thermocycler SimpliAmp Thermal Cycler (Applied Biosystems, Thermo Fisher, Waltham, MA, USA), and the products were separated by capillary electrophoresis on an ABI-3500 genetic analyzer (Applied Biosystems, Thermo Fisher, Waltham, MA, USA). The resulting peak intensities were analyzed using Coffalyser software (https://www.mrcholland.com/technology/software/coffalyser-net) (MRC-Holland) which performed the intrasample and intersample normalization of the peaks with the manufacturer’s reference probes and normal control DNA, respectively. Values above 1.3 were considered as gain, between 1.3 and 0.75 normal, between 0.75 and 0.25 heterozygous loss, and below 0.25 homozygous loss. 

### 2.5. Sanger Sequencing for Detection of Germline PAX5 Variants

The polymerase chain reaction (PCR) primers (see Supplementary Table S2) were designed according to the established laboratory protocol. The reference sequence for each amplicon was acquired from the ENSEMBL database. Optimal primers were selected by the Primer3 online tool (v. 0.4.0) [29] and analyzed for possible polymorphisms with the SNPcheck tool [30]. 

The primers from the PCR amplification reaction were removed in the first step with the use of the ExoSap-IT enzyme mix (Affymetrix, Santa Clara, CA, USA), followed by sequence reaction with the BigDye Terminator sequencing master mix (Applied Biosystems, Waltham, MA, USA) according to the established laboratory protocol. The sequencing was performed on the ABI-3500 genetic analyzer (Applied Biosystems, Thermo Fisher, Waltham, MA, USA). Sequences were aligned to the reference sequence (NG_033894.1) from the NCBI Reference Sequences and analyzed with the BLAST software 2.15.0 (https://blast.ncbi.nlm.nih.gov/Blast.cgi, accessed on 31 August 2023).

### 2.6. Statistical Analysis

Comparisons of categorical values were carried out using the logistic regression and Fischer’s exact test, with the use of Bonferroni correction to adjust for multiple testing. For the comparison of numerical values, the Mann–Whitney U-test was used. Survival analysis was carried out with the Kaplan–Meier method, and Cox regression models were used for testing the significance of variables on the outcomes. To adjust for small sample sizes in survival analysis within individual genetic subtypes, we performed the permutations test on the log-rank statistic with the R package FHtest. The significance level for all the tests was 5% (*p* < 0.05 was considered to be statistically significant). An event was defined as failure to achieve remission, a relapse after remission, the development of a second malignant neoplasm, or death. Overall survival was defined as time to death. Both event-free survival (EFS) and overall survival (OS) were censored at last contact. 

## 3. Results

### 3.1. PAX5alt and PAX5 P80R Patients

Among our cohort of 99 patients, we found eight patients with *PAX5*-associated genetic subtypes (Table 1). We identified six patients with the PAX5alt signature and one with the PAX5 P80R subtype with the tool ALLCatchR. The classifier MD-ALL identified one additional sample as PAX5alt which was classified as Ph-like by ALLCatchR. Manual inspection of the fusion transcripts and variants showed no compelling evidence for Ph-like genetic alterations. We did, however, identify a missense variant in *PAX5* with computational prediction tools unanimously supporting a deleterious effect on the gene. Therefore, this sample was regarded as PAX5alt. For seven of these patients, cytogenetic results were available, and for all of them, RT-PCR data were available. All of these patients were previously classified as B-other as they did not exhibit any recurrent abnormalities (*BCR::ABL1*, *ETV6::RUN1*, *TCF3::PBX1*, *KMT2A*, hyperdiploidy, hypodiploidy). The PAX5alt subtype therefore accounted for 7.1% of our whole B-ALL cohort and 29.1% of the B-other group, while the one patient with PAX5 P80R represented 1.0% of the whole cohort and 4.2% of the B-other group. One sample with the *BCR::ABL1* fusion gene was classified as PAX5alt by both prediction tools but was manually assigned to the BCR::ABL1 subgroup.

### 3.2. Genetic Changes Identified in PAX5alt and PAX5 P80R Patients

We found diverse genetic alterations in our patients with the PAX5alt gene expression profile (Table 1). We identified four patients with rearrangements in *PAX5*. Two patients carried the fusion gene *PAX5::FKBP15*, and two had the fusion gene *PAX5::FOXP1*. Furthermore, two patients carried a missense single nucleotide variant in *PAX5*. The first had a substitution in exon 2, c.101C>T, leading to an amino acid change from proline to leucine at position 34, which was classified as likely pathogenic according to the ACMG guidelines. The second carried a substitution in exon 6, c.725A>G, changing the glutamic acid at position 242 to glycine, classified as a variant of uncertain significance (VUS). One patient had a frameshift variant, a deletion of four nucleotides in exon 9, c.1045_1048del, leading to a stop codon after 53 amino acids, and a partial loss of the C-terminal domain. This variant was classified as likely pathogenic and explained the predicted PAX5alt subtype. Germline samples were available for these three patients, and we did not confirm the presence of these variants in their samples. We also identified one patient with the recurrent *PAX5* P80R somatic variant without any CNVs that we investigated. We did, however, identify this patient as a compound heterozygote with three missense variants on the second allele that were not expressed. These were variants in exons 5 (c.596G>A, p.R199K), 7 (c.826G>A, p.D276N) and 10 (c.1102T>A, p.S368T), all classified as VUS.

MLPA results revealed that five of the seven PAX5alt patients carried additional deletions in *PAX5*, leading to the loss of heterozygosity, of which two patients had additional deletions present and were therefore classified as *IKZF1*^plus^. One patient had an amplification in *PAX5*. All patients classified as PAX5alt also carried deletions in *CDKN2A* and *CDKN2B*. 

Considering the other single nucleotide variants in these patients, we identified on average 17 additional cancer-related variants per patient in 43 different genes in patient samples with the PAX5alt and PAX5 P80R subtypes, the most commonly altered genes being *ATM, ATRX, CYP2D6, CYP2D7, KDM5A, KMT2A,* and *NCOR1* (see Supplementary Table S3). 

### 3.3. Clinical Characteristics and Outcomes for Patients with the PAX5alt and PAX5 P80R Subtypes

**Figure 1:**
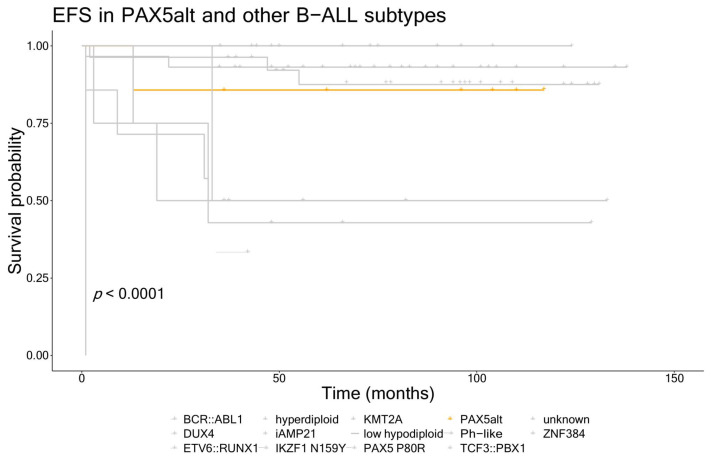
Event-free survival for patients with PAX5alt vs. other B-ALL subtypes in our cohort (PAX5alt subtype is marked in orange).

Most of the patients with the PAX5alt and PAX5 P80R subtypes were male (75%) and only one was initially classified as standard risk; however, the difference between this group and other B-ALL patients from our cohort was not statistically significant (Table 2). They also did not differ from other B-ALL cases in other clinical characteristics (WBC at diagnosis, blast cell count on day 8, MRD on days 15 and 33, CNS involvement, age at diagnosis). One patient (14.3%) in the PAX5alt group and seventeen patients (18.7%) in the rest of the B-ALL cohort experienced an event. When comparing the PAX5alt and PAX5 P80R patients to the rest of the cohort, event-free survival analysis also showed no differences between the groups (5-year EFS 88% vs. 84%, respectively). A similar trend was observed for overall survival, with one (14.3%) patient in the PAX5alt group and six (6.6%) patients in B-ALL other group that died (5-year OS 88% vs. 93%, respectively). However, when putting the EFS in the context of all of the B-ALL genetic subtypes in our cohort, we can see the patients with PAX5alt had intermediate outcomes compared to other subtypes (Figure 1).

Taking a closer look at the two patients with the *PAX5::FKBP15* fusion, both were male and CNS negative, but presented with a high WBC at diagnosis. One presented with a complex karyotype with a small hypodiploid clone and was therefore classified as high risk. However, his MRD was negative on days 15 and 33, and the rest of the treatment was without complications despite the presence of the *IKZF1*^plus^ profile. On the other hand, the second patient was a good prednisone responder, but the day 15 MRD was positive, so he was classified as intermediate risk. MRD on day 33 was negative. MLPA results showed a partial deletion in *PAX5*. Later, this patient experienced a very early relapse with a monocytic switch after 13 months. This resulted in a rapid progression of the disease and death. Subsequent analysis showed that this patient carried a somatic pathogenic variant in *PTPN11* (*PTPN11*:c.211T>C). We could not confirm the presence of this variant at the time of relapse as there was not sufficient material available.

### 3.4. PAX5 Variants in Other B-ALL Subtypes

One patient, who was otherwise classified as BCR::ABL1-positive, carried a secondary variant in *PAX5* (c.197G>A, p.S66N, classified as VUS) alongside a deletion, leading to the loss of heterozygosity. This was the sample that was assigned to the PAX5alt signature by the two gene expression prediction tools. No other secondary *PAX5* variants or gene fusions were identified in any of the patients that were not classified in the PAX5alt or PAX5 P80R subtypes. 

### 3.5. PAX5 CNVs and Their Clinical Significance

For seven patients out of the entire cohort (7%), we did not have data from the MLPA analysis. Of the remaining 92, 31 (33.7%) carried CNVs in *PAX5*. In our cohort, neither deletions nor amplifications in *PAX5* were notably predominant. Specifically, 17 of the patients with the CNVs were found to have deletions, while duplications were observed in 14 patients. The deletions were all heterozygous, the most common being the deletion of the whole gene, followed by different partial deletions. The most common were amplifications in exon 7 and/or 8 which were present in eight of the fourteen patients with duplications, followed by amplifications of the whole gene as a result of hyperdiploidy (four patients). 

**Figure 2:**
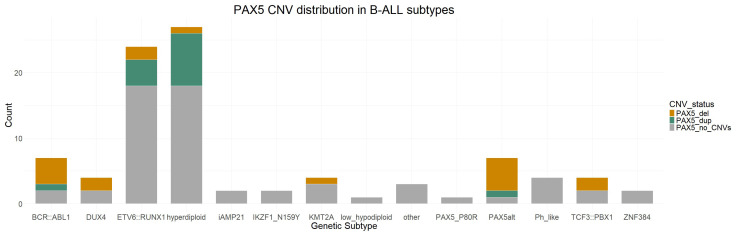
Frequency of *PAX5* CNVs in B-ALL genetic subtypes.

**Figure 3:**
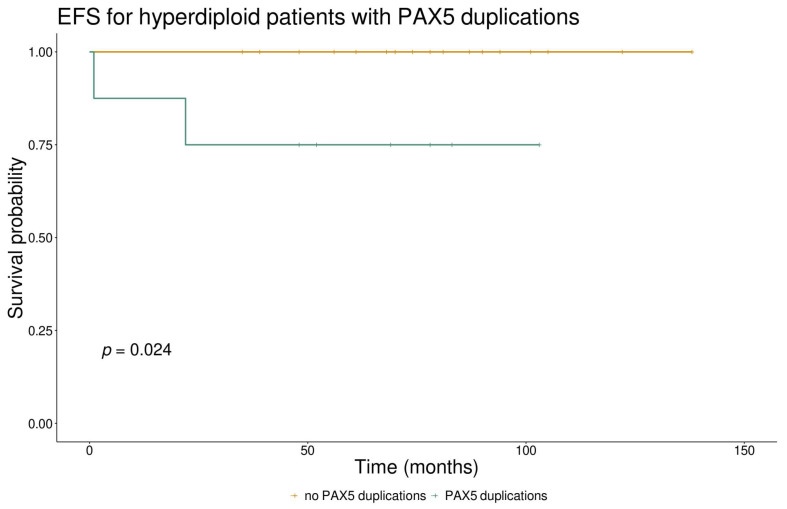
Event-free survival for hyperdiploid patients with *PAX5* duplications vs. those without *PAX5* duplications.

CNVs in *PAX5* were significantly enriched in males compared to females (*p* = 0.015) but not in any specific genetic subtype despite more than half of the patients with the PAX5alt and BCR::ABL1 genetic profiles carrying them (Figure 2). Two subtypes stood out with a significantly lower incidence of *PAX5* deletions, namely ETV6::RUNX1 (*p* = 0.011) and hyperdiploid cases (*p* = 0.005). The presence of any *PAX5* CNVs was not shown to be associated with survival outcomes or other clinical parameters when looking at the whole cohort. We further investigated the duplications in *PAX5* in hyperdiploid and ETV6::RUNX1-positive cases as they were abundantly present in these genetic subtypes (29.6% of hyperdiploid and 17% of ETV6::RUNX1 patients). Hyperdiploid cases carrying *PAX5* duplications had significantly higher MRD values at the end of induction (*p* = 0.017) compared to those without the duplications. We also observed poorer event-free survival in hyperdiploid cases carrying *PAX5* duplications compared to those without, with 5-year EFS of 75% vs. 100%, respectively (*p* = 0.024) (Figure 3); however, the same effect was not observed in patients with the ETV6::RUNX1 subtype (*p* = 0.371). 

## 4. Discussion

Our aim was to investigate the frequency and types of genetic alterations in *PAX5* in our cohort of 99 children with B-ALL. As expected, *PAX5* was impacted in a high percentage of our patients (33.3%), with the genetic alterations ranging from CNVs and rearrangements to SNVs. The most common were CNVs, which were found in more than a third of the patients, followed by point mutations in 5.2%, and gene rearrangements in 4.1%. 

The reports about the frequencies of the recurrent B-ALL genetic subtypes associated with *PAX5* (PAX5alt and PAX5 P80R) show that they occur in 7.0–10.0% and 0.2–4.0% of childhood patients, respectively [7,31,32,33]. These frequencies were very similar in our cohort, with PAX5alt and PAX5 P80R representing 7.1% and 1.0% of all patients, respectively. With the current knowledge of these subtypes, there are conflicting reports regarding outcomes for these patients. While some reports state that both subtypes are associated with intermediate to favorable outcomes, the subject matter seems to be more diverse. In adult B-ALL cases, PAX5 P80R has been associated with favorable outcomes [34], while it has been shown to contribute to poorer outcomes in childhood cases [31]. The PAX5alt subtype is associated with intermediate outcomes [35], and patients with this subtype are reported to be classified as high risk more often than standard risk, according to National Cancer Institute criteria [36]. The event-free and overall survival of the patients with alterations in *PAX5* did not differ from the rest of the patients in our cohort (Figure 1), with the 5-year EFS being 88% and 84%, respectively, and 5-year OS being 88% and 93%, respectively. When looking at the EFS within the distinct genetic subtypes, however, we notice that PAX5alt patients seem to have intermediate outcomes in our cohort as well, and they were in most cases classified as non-SR. 

CNVs in *PAX5* are present in around 30% of all B-ALL patients [5], the most common being heterozygous deletions leading to haploinsufficiency, as was also the case in our cohort. Confirming the high incidence of these CNVs, we also showed that they are more often found in males compared to females but are not significantly associated with clinical outcomes in patients, corroborating the findings that they are not considered predictive in the context of MRD levels and are not independently indicative of poor prognosis in childhood B-ALL [37]. We took a closer look at the CNVs within some genetic subgroups, and showed a trend of poorer survival in hyperdiploid cases carrying duplications in *PAX5* compared to other hyperdiploid cases. This poorer survival did not seem to be influenced by other factors such as sex, age, or the presence of other CNVs; therefore, it could be indicative of a more complex interplay of genetic factors within a genetic subtype that is otherwise considered standard risk. However, as our results arise from a small cohort, additional research should be conducted and validated on a larger cohort with the possibility to better account for confounding variables. Additionally, many of the duplication cases we had in this cohort were duplications of exons 7 and/or 8, and they were mostly present in ETV6::RUNX1-positive and hyperdiploid cases, which has not been described in other study cohorts. There have been reports of recurrent intragenic amplifications of *PAX5* involving exon 2 or exon 5 or both of these exons [38,39], which are found in patients that lack other major cytogenetic abnormalities and are associated with poorer outcomes. Interestingly, we did not identify any cases like this in our cohort. 

Pathogenic variants in *PAX5* are less common and are found in around 7% of the patients [5,40]. The consequence of these variants is the creation of a non-functional protein that loses its ability to bind to DNA or to regulate transcription. These variants are rarely germline; however, some cases have been described [8,9,41]. We identified *PAX5* variants in five (5.2%) patients, of which one carried the recurring variant p.P80R, three were classified as PAX5alt, and one was BCR::ABL1-positive. Among the PAX5alt patients, two had a missense variant and one a frameshift deletion. One of the missense variants (c.101C>T), which affects the paired box domain, has previously been described as somatic in B-ALL patients [42], and one case with this variant has been reported in intestinal adenocarcinoma [43]. The other missense variant (c.725A>G) in the partial homeodomain and the frameshift deletion (c.1045_1048del) in the C-terminal domain have not yet been described in literature or reported in the COSMIC database. One patient had a secondary variant in this gene (c.197G>A) with the deletion of the second allele, while also carrying the *BCR::ABL1* fusion gene. This variant has been reported in one sporadic case of B-ALL [9]. This sample was also classified as PAX5alt according to the gene expression profiling classifiers, indicating a strong influence of the *PAX5* alterations on the global gene expression and processes in the cell. None of the variants we found were present in the germline samples we tested, confirming their somatic origin. 

In previous studies, most patients with PAX5 P80R were compound heterozygotes [31], and this was also true in our case. In addition to the variant c.239C>G, this patient harbored three missense variants on the second allele, which was not expressed. Two variants (c.596G>A and c.826G>A) have previously been reported in malignant melanoma, bladder cancer, and adenocarcinoma [44,45], while the third (c.1102T>A) has not been described in the literature yet. The *PAX5* p.P80R variant has also been shown to be a somatic and not a germline event [31,46], as we also confirmed for our patient who experienced no notable complications during the whole treatment period.

Translocations involving the *PAX5* gene are observed in approximately 2.5% of pediatric and 1% of adult patients with B-ALL [4,7]. These translocations often result in the formation of chimeric genes that encode proteins preserving the DNA-binding paired box domain and the nuclear localization signal of PAX5, but with C-terminal domains derived from the fusion partners. Various partner genes, including transcription factors, structural proteins, and signal transducers, have been identified as fusion partners with the *PAX5* gene [7,12]. We found *PAX5* rearrangements in four patients, all classified as PAX5alt. In two patients, the fusion partner was *FKBP15*, and in the other two, *PAX5* fused to *FOXP1*, both of which have already been described as fusion genes in B-ALL [5,10,47,48,49]. None of the Ph-like patients in our cohort carried any translocations involving this gene despite *PAX5* translocations being very common in this subgroup of patients [7,11,12]. 

Among our patients with altered *PAX5*, we also report an interesting case of a patient with the *PAX5::FKBP15* fusion gene who underwent a monocytic switch. Several cases of a switch from ALL to AML have been described, with the most frequent subtypes that undergo the switch being DUX4-rearranged, PAX5 P80R, and ZNF384-rearranged [50,51]. To our knowledge, this is the first described case of a PAX5alt patient undergoing a monocytic switch, further showing the complexity and interplay of genetic factors in this disease. This patient carried a somatic pathogenic variant in *PTPN11*. This variant, associated with myeloproliferative diseases, was already present at the time of the first diagnosis of ALL. *PTPN11* encodes the Shp2 non-receptor protein–tyrosine phosphatase implicated in several signaling pathways. Activating mutations in *PTPN11* are most commonly associated with juvenile myelomonocytic leukemia but are not as well defined in other neoplasms. They have been reported at a rate of 6.6% in AML [52,53] and some cases of high hyperdiploid ALL [54], with this particular variant mainly described in JMML and some AML cases. It is important to note that one of the main functions of PAX5 is maintaining non-B-cell genes repressed during B-cell differentiation; therefore, B-ALL tumors lacking PAX5 function may express lineage promiscuity markers such as monocytes without implying a lineage switch [55]. Nevertheless, identifying pathogenic variants in genes associated with myeloproliferative diseases might, therefore, aid in the identification of patients who are at high risk of a monocytic switch. 

## 5. Conclusions

In summary, this study contributes valuable insights into the presence, frequency, and prognostic implications of diverse *PAX5* alterations in B-ALL patients, underscoring the complex interplay of genetic factors and their impact on patient prognosis. Our findings reveal a high frequency of *PAX5* alterations, the most common being CNVs, which may hold additional prognostic information within specific genetic subtypes, followed by single nucleotide variants and fusion genes that are in most cases the oncogenic drivers, particularly in PAX5alt and PAX5 P80R subtypes. Recognizing these alterations within routine diagnostic frameworks is crucial, especially in the anticipation of forthcoming investigations aimed at tailoring therapeutic approaches for these patients to improve clinical outcomes.

## Figures and Tables

**Table 1:** Genetic and clinical characteristics of B-ALL patients with a *PAX5*-associated genetic subtype.

	Patient Nr.							
	1	2	3	4	5	6	7	8
Predicted subtype	PAX5alt	PAX5alt	PAX5alt	PAX5alt	PAX5alt	PAX5alt	PAX5alt	PAX5 P80R
Genetic variants in *PAX5*	/	/	/	c.1045_1048del	c.101C>T	/	c.725A>G	[c.239C>G]; [c.596G>A, c.826G>A, c.1102T>A]
Protein change	/	/	/	p.P349Sfs*53	p.P34L	/	p.E242G	p.P80R, p.R199K, p.D276N, p.S368T
ACMG classification	/	/	/	LP	LP	/	VUS	LP, VUS, VUS, VUS
Fusion genes involving *PAX5*	*PAX5::FKBP15*	*PAX5::FOXP1*	*PAX5::FOXP1*	*/*	*/*	*PAX5::FKBP15*	*/*	*/*
CNVs	*IKZF1* del, *CDKN2A/2B* del., *PAX5* ex. 10 del. (*IKZF1*^plus^)	*CDKN2A/2B* del.	*PAX5* del., *CDKN2A/2B* del.	*PAX5* del., *CDKN2A/2B* del.	*PAX5* ex. 8 dup., *CDKN2A*/*2B* del.	*PAX5* ex. 8 and 10 del., *CDKN2A*/*2B* del.	*IKZF1* del., *PAX5* del., *CDKN2A*/*2B* del. (*IKZF1*^plus^)	/
Gender	male	female	female	male	male	male	male	male
Age at diagnosis (years)	4	7	4	1	19	3	3	8
WBC at diagnosis (×10^9^/L)	22.6	8.7	1.1	17.2	5	32.2	372.3	1.98
CNS involvement	neg.	neg.	neg.	pos.	neg.	neg.	neg.	neg.
Day 8 prednisone response (blasts/mm^3^)	280	1750	45	78	0	0	1078	0
MRD day 15 (%)	0	50	0.019	0.6	0.01	1.17	0.43	0
MRD day 33 (%)	0	0	0	0.016	0	0	0	0
End of induction risk classification	HR	HR	SR	IR	IR	IR	IR	IR
Event	no	no	no	no	no	relapse with AML switch, death	no	no

(LP—likely pathogenic; VUS—variant of uncertain significance; ex.—exon; del.—deletion; dup.—duplication; neg.—negative; pos.—positive; SR—standard risk; IR—intermediate risk; HR—high risk; CNVs—copy number variations; WBC—white blood count; CNS—central nervous system; MRD—minimal residual disease).

**Table 2:** Patients’ clinical characteristics.

**Patient Characteristics**	**PAX5alt or PAX5 P80R (*n* = 8)**	**Other (*n* = 91)**
Gender		
male	6	48
female	2	43
Age at diagnosis		
˂1 year	0	3
1–5 years	5	52
≥6 years	3	36
WBC at diagnosis		
˂20 × 10^9^/L	5	71
≥20 × 10^9^/L	3	20
CNS involvement		
yes	1	6
no	7	83
unknown	0	2
Day 8 prednisone response		
˂1000 blasts/µL	6	79
≥1000 blasts/µL	2	10
unknown	0	2
MRD day 15		
˂0.1%	4	31
0.1–10%	3	45
˃10%	1	12
unknown	0	3
MRD day 33		
˂0.01%	7	66
≥0.01%	1	22
unknown	0	3
End of induction risk classification		
standard	1	16
intermediate	5	54
high	2	21

## Data Availability

The data presented in this study are available on request from the corresponding author. Hardcopies of all data and results are also available in patients’ files and the laboratory that conducted the tests. The data are not publicly available due to privacy and ethical restrictions.

## Supplementary Materials

The following supporting information can be downloaded at: https://www.mdpi.com/article/10.3390/cancers16061164/s1, Table S1: UCSF500 Cancer Gene Panel; Table S2: Primer sequences for exons of PAX5; Table S3: Variants identified in PAX5alt and PAX5 P80R patients from RNAseq data.
